# The relationship between objective and subjective cognitive performance and clinical and MRI disease burden in early multiple sclerosis

**DOI:** 10.1038/s41598-026-48645-6

**Published:** 2026-05-16

**Authors:** Delphine Van Laethem, Lars Costers, Augusto Miravalle, Enrique Alvarez, Bennett Myers, Aaron Boster, Hanan Al Halawani, Maarten Dewil, Melissa Cambron, Matthias Grothe, Stijn Denissen, Rebecca Bartz, Diana Sima, Annemie Ribbens, Dirk Smeets, Guy Nagels

**Affiliations:** 1 AIMS Lab, Vrije Universiteit Brussel/UZ Brussel, Brussels, Belgium; 2 Department of Physical and Rehabilitation Medicine, Universitair Ziekenhuis Brussel, Brussels, Belgium; 3 icometrix, Leuven, Belgium; 4 Rush University, Chicago, IL USA; 5 Rocky Mountain Multiple Sclerosis Center, University of Colorado, Aurora, CO USA; 6 DENT Neurologic Institute, Amherst, NY USA; 7 The Boster Center for Multiple Sclerosis, Columbus, OH USA; 8 Texas Neurology, Dallas, TX USA; 9 Department of Neurology, Imelda Hospital, Bonheiden, Belgium; 10 AZ Sint-Jan Brugge, Brugge, Belgium; 11 University of Ghent, Ghent, Belgium; 12 Universitätsmedizin Greifswald, Greifswald, Germany; 13 Neurology Department, Universitair Ziekenhuis Brussel, Brussels, Belgium; 14 St. Edmund Hall, University of Oxford, Oxford, UK

**Keywords:** Diseases, Health care, Neurology, Neuroscience

## Abstract

Subjective cognitive performance in multiple sclerosis (MS) correlates weakly with objective performance, but is more strongly associated with depression. We aimed to identify symptoms and brain MRI volumes related to subjective and objective cognitive performance. 205 MS subjects, diagnosed within the last 15 years, completed the SymptoMScreen, patient-reported Expanded Disability Status Scale, Neuro-QoL subjective cognitive and fatigue sub-scores, and Beck Depression Inventory. For the objective cognitive assessment the smartphone-based icompanion Symbol test was used. Volumetric variables were calculated from the 52 available brain MRIs using icobrain. A weak correlation was observed between subjective and objective performance (rho = 0.21, *p* = 0.002). Subjective performance was negatively associated with pain, dizziness, fatigue and depression, while objective performance was negatively related to walking impairment. Objective performance significantly correlated with thalamic volume, while subjective performance did not correlate with any brain volumes. In other words, subjective and objective cognitive performance are related to different clinical markers: subjective performance is linked to invisible symptoms, while objective performance is linked to more visible measurable clinical markers. Integrating both perspectives in clinical practice may provide a more holistic understanding of cognition in MS, allowing for tailored interventions that enhance patient care and quality of life.

## Introduction

Around 2.8 million people worldwide suffer from multiple sclerosis (MS), the most frequent neuroinflammatory and neurodegenerative disease in young adults^1^. Between 34 and 65% of patients suffer from cognitive impairment, most frequently in the domains of cognitive processing speed, learning and memory^2^. Furthermore, recent studies show that naming and word-finding difficulties are also commonly seen^3,4^. Cognitive performance is objectified using targeted and sensitive neuropsychological tests, such as the Symbol Digit Modalities Test (SDMT)^5,6^. In recent years there has been an increasing interest in digitalization of these assessments, which allows for more ease of administration without the need for trained personnel and extraction of potential additional metrics that are more difficult to capture using traditional paper-pencil tests (such as fatigability)^2,7^.

Due to lack of time and trained personnel, clinicians often have to rely on patient-reported cognitive symptoms as a first screening tool^5^. Several patient-reported questionnaires have been developed to assess perceived cognitive symptoms in MS^8–10^, while some studies use cognitive sub-scores of quality of life questionnaires^11–14^ or visual analogue or Likert scales^15,16^ to assess subjective cognition. However, patient-report cognitive performance measures are consistently only weakly associated with objective measures, while correlating more strongly with depression^8,13,14,16–23^ and fatigue^16,23,24^. It is unclear what drives this disparity; some have suggested that subjective cognitive impairment may precede objective impairment and thus reflect subtle changes in cognitive performance that do not yet meet the threshold for impairment in objective neuropsychological tests^25,26^. Others propose that subjective cognitive symptoms solely reflect underlying depression^20^.

Nevertheless, studies have shown an association between objective and subjective measures of cognition when controlling for depression^21,25^. Furthermore, Julian et al. found that after successful treatment for depression subjective cognitive performance was more strongly related to objective impairment, while depression was no longer a significant predictor^14^. Subjective cognitive performance has also been related to reduced vitality, health-related quality of life, social network satisfaction^27^, reduced health-promoting behaviours^12,19,27^, unemployment^15,27,28^, physical comorbidities^19^, reduced thalamic and cortical grey matter volumes^11^ and reduced lower total hippocampal and CA1 volumes^26^. Finally, while subjective cognitive impairment has been related to relapsing-remitting MS and people with a progressive disease are more likely to overestimate their cognitive performance^22^, the relationship between subjective and objective scores was not found to be influenced by the patient’s level of physical disability^16,21^. To the best of our knowledge, no studies have directly compared how different clinical characteristics and MRI volumes relate to subjective versus objective cognitive performance.

In this study we aim to identify the difference in factors related to subjective and objective cognitive performance, including MS symptoms, disability, fatigue and depression. Furthermore, we assess the correlations between subjective and objective cognitive performance and brain MRI volumes.

## Methods

### Study sites & participants

Three hundred persons with clinically isolated syndrome (CIS) or clinically definite relapsing-remitting MS (RRMS), as defined by the revised McDonald criteria^29^, were recruited in 9 clinical centres in Belgium (Imeldaziekenhuis Bonheiden, AZ Sint-Jan Brugge, and UZ Brussel), Germany (Universitätsmedizin Greifswald) and the United States of America (DENT Neurologic Institute, UCHealth University of Colorado Hospital, Texas Neurology, The Boster Center for Multiple Sclerosis, and Advanced Neurosciences Research Colorado). The inclusion criteria were: age between 18 and 65 years old, absence of hand function problems which limit the use of a smartphone, no history of relapse with onset 30 days prior to start of study, no other major neurological or psychiatric disorders, and no history of cognitive rehabilitation treatment. Exclusion criteria were a disease duration of longer than 15 years (to focus on patients with ‘early MS’), established cognitive disorders other than MS, and known drug and/or alcohol abuse. Of those 300 subjects, 3 were excluded from analysis due to incomplete data and 92 discontinued the study, leading to a total of 205 subjects suitable for further analysis.

This study was conducted in accordance with the ethical principles that have their origin in the Declaration of Helsinki and in accordance with Good Clinical Practices (GCP). The experimental protocols were approved by institutional and/or licensing committees, more specifically the Commissie Medische Ethiek Imeldaziekenhuis Bonheiden, Commissie voor Ethiek AZ Sint-Jan Brugge and Commissie Medische Ethiek UZ Brussel for the participating centers in Belgium, the Ethikkommission Universitätsmedizin Greifswald for the center in Germany and the Institutional Review Board Advarra for the centers in the United States of America. Freely given written informed consent was obtained from each subject, or, in those situations where consent could not be given by the subject, from their legally acceptable representatives, prior to study participation.

### Data collection and outcome measures

All data was collected using the icompanion app^30^, an FDA registered and CE-marked (level IIa) medical device, of which a study-specific version (which includes the outcome measures mentioned here) was installed on the participants’ smartphone. Participants were asked to complete patient-reported outcomes (PROs) and an objective cognitive assessment at home (remote, non-clinical setting), at a single cross-sectional timepoint (30–45 min in total). The PROs included the SymptoMScreen for assessment of MS symptoms^31,32^, patient-reported Expanded Disability Status Scale score for disability (prEDSS^33^), Neuro-QoL sub-scores for subjective cognitive impairment and fatigue(^34^, t-scores compared to reference population^35^), and the Beck Depression Inventory (BDI) for depression^36^. For the objective cognitive assessment, the validated smartphone-based cognitive screening battery icognition^7^ was used. This battery includes the Symbol Test (information processing speed), the Dot Test (visuospatial short-term memory and learning) and the visual Backwards Digit Span (visual working memory). In the validation study^7^, the Symbol Test showed the best results on all validation criteria. Since the Symbol Digit Modalities Test (SDMT), on which the Symbol Test is based, is considered the gold standard for cognitive assessment in MS^2,5^, we only included the Symbol Test in the analyses of this paper. The scores for the Symbol test were based on the total number of correct trials in 90 s. For the cognitive and fatigue sub-score of the Neuro-QoL and the Symbol test, a higher score indicates a better performance or less symptoms/limitations. The opposite is true for the SymptoMScreen, prEDSS and BDI.

Furthermore, the following demographic and clinical information was collected: age, sex, disease duration, disease type (CIS or RRMS) and disability as measured by the Expanded Disability Status Scale (EDSS^37^). Finally, local study teams uploaded brain MRIs (if available) from the last six months before signing the informed consent, using the icompanion web portal.

### MRI volumetry

MRI volumetric variables were calculated using the ico**brain** software-assisted MRI measurement tool^30,38^, which is the commercial volumetric MRI reporting tool in MS with the most validation studies^39^. The following volumetric parameters were analysed: black holes, caudate, cortical grey matter (frontal lobe, occipital lobe, parietal lobe, temporal lobe), cerebrospinal fluid (CSF), deep white matter lesions, grey matter, hippocampus, infratentorial lesions, juxtacortical lesions, lateral ventricles, periventricular lesions, thalamus, and whole brain volume. To optimize inter-subject comparisons, we opted to use brain volumes normalized for head size with respect to an MNI atlas. In contrast, lesion and black hole volumes were intentionally left unnormalized, as these represent focal pathological processes rather than global anatomical variability. Normalization to head size could obscure clinically meaningful differences by scaling lesion burden relative to intracranial volume, which is not biologically relevant for quantifying absolute tissue damage. Given that lesions and black holes reflect cumulative, discrete areas of demyelination and axonal loss, expressing them in absolute volumes (e.g., mL) preserves their pathological significance and better aligns with clinical interpretation and prognostic utility. This approach ensures that lesion burden is not underestimated in individuals with larger brain sizes, thereby maintaining the integrity of the structure-function correlations under investigation.

Since the source data originates from different sites, all using their respective clinical acquisition protocols, some heterogeneity in scan quality was observable. To ensure a high standard of biomarker quality, we opted to process data according to its image quality. When both a high quality T1-weighted and FLAIR-scan was available, all brain volumes, substructures and lesion volumes were calculated. If the T1-sequence had a suboptimal (low) resolution, substructures were discarded and only whole brain volume was calculated. If the FLAIR-scan was suboptimal, we opted to focus only on total lesion volume, rather than also stratifying it into the stereotypical brain regions for MS. Of note, a handful of scans only contained a ‘black blood’ T1-sequence, which interfered with correct brain segmentation and therefore led to unreliable brain volumes. Here we opted to only provide lesion volumes, as these segmentations were still very adequate.

### Statistics

First, the relationship between subjective and objective cognitive performance was assessed using a Spearman correlation, since the Neuro-QoL Cognitive sub-score was not normally distributed (Shapiro’s test: W = 0.98, p-value = 0.005). Next, subjective and objective cognition scores were dichotomized into impaired (a score worse than 1.5 standard deviation from the reference population) or non-impaired. For the Neuro-QoL Cognitive sub-score, this was based on a US General reference sample(^35^, cut-off score: 35), and for the Symbol test score, this was based on data from 82 healthy controls(^7^, cut-off score: 15.8). Then a confusion matrix was calculated.

Second, to identify the difference in clinical characteristics related to subjective and objective cognitive performance, we used multiple regression models with either subjective or objective cognitive as the dependent variable, and the following independent variables: MS symptoms (SymptoMScreen), fatigue, prEDSS, depression. SymptoMScreen Fatigue, SymptoMScreen Cognitive and SymptoMScreen Depression were excluded due to overlap with the more extensive questionnaires/assessments Neuro-QoL Fatigue, icognition Symbol test/NeuroQoL Cognitive and BDI.

Finally, the correlation between the MRI volumetric parameters and objective and subjective cognitive performance was assessed using Spearman correlations, after which p-values were corrected using FDR correction^40^.

A two-sided test with a type I error probability of 0.05 was used for all analyses.

## Results

### Demographic & clinical information

The demographic and clinical information is listed in Table 1.

**Table 1:** Demographic and clinical information.

	Overall (*n* = 205)
Age (years), mean ± SD	40.41 ± 9.55
Gender, % Female	71.71%
Type of MS, %	0.5% CIS, 95.5% RRMS
Disease duration (years), mean ± SD	5.71 ± 4.22
EDSS, median (interquartile range)	2 (1)

SD = standard deviation.

### Relationship between subjective and objective cognitive performance

**Fig. 1:**
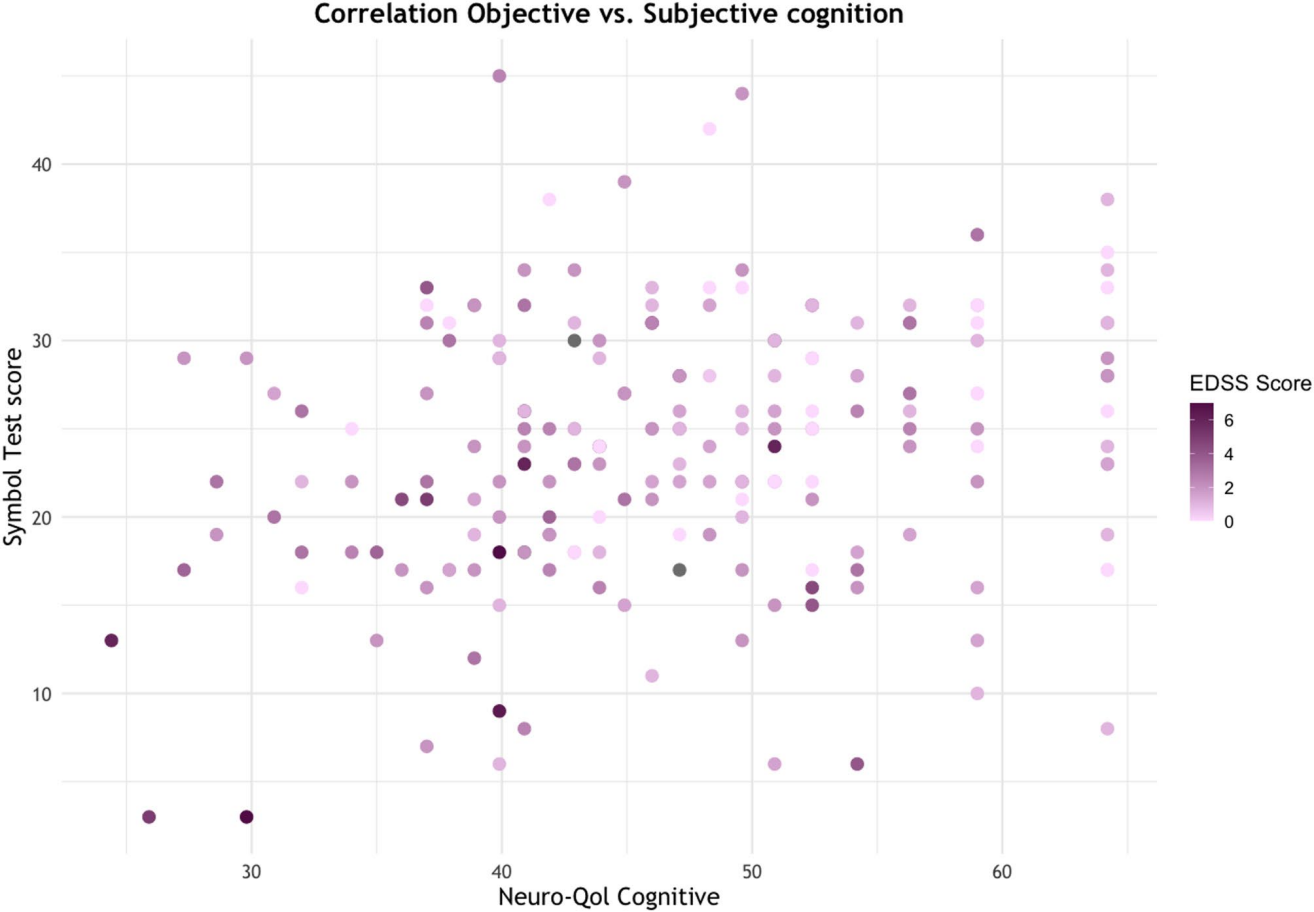
Scatterplot of the association between the Neuro-QoL Cognitive sub-score and the Symbol test for objective cognition. The EDSS score of each subject is depicted using a colour scale.

There was a significant positive correlation (rho = 0.21, *p* = 0.001) between Neuro-QoL Cognitive sub-score (subjective cognitive performance) and the Symbol test score (objective cognitive performance). This relationship is depicted using a scatter plot in Fig. 1. The EDSS score of each subject is depicted using a colour scale.

In order to better understand the relationship between subjective and objective cognitive performance, a confusion matrix was calculated based on whether subjects were impaired or not impaired on the two measures (see Table 2).

**Table 2:** Confusion matrix of NeurQoL Cognitive sub-score and Symbol test. Impairment was defined as a score of at least 1.5 standard deviations lower than a healthy reference population.

	Symbol test		
	Impaired	Non-impaired	Total
Neuro-QoL Cognitive sub-score			
Impaired	2.0%	*7.5%*	9.5%
Non-impaired	8.0%	82.5%	90.5%
Total	10.0%	90.0%	

### Co-variates influencing subjective and objective cognitive performance

#### Subjective cognitive performance

We used a multiple linear regression model to investigate the relationship between the dependent variable subjective cognitive performance (Neuro-QoL Cognitive sub-score), and independent variables related to MS symptoms (SymptoMScreen: walking, hand function, spasticity, pain, sensory, bladder, vision, dizziness, anxiety), fatigue (Neuro-QoL Fatigue), disability (patient-reported EDSS), and depression (Beck’s depression inventory; BDI). A check for multicollinearity was performed using the variance inflation factor (VIF). The VIF values for the predictor variables were all within acceptable limits, indicating no severe multicollinearity issues.

The initial model (Table 3) included all the predictor variables, and showed that pain (β = -1.41, *p* = 0.003), Neuro-QoL Fatigue (β = -0.27, *p* = 0.003) and depression (β = -0.23, *p* = 0.001) had a statistically significant negative association with the Neuro-QoL Cognitive sub-score.

**Table 3:** Results of multiple regression model with subjective performance as dependent variable.

Predictor	Estimate	Std. error	t-value	*p*-value
Intercept	*71.29*	*3.71*	*19.22*	***< 2e − 16***
Walking	− 0.27	0.59	− 0.46	0.65
Hand function	0.13	0.53	0.24	0.91
Spasticity	0.20	0.49	0.40	0.69
Pain	*− 1.41*	*0.47*	*− 3.02*	***2.90e − 3***
Sensory	− 0.27	0.42	− 0.65	0.52
Bladder	− 0.38	0.38	− 1.00	0.31
Vision	0.01	0.37	0.03	0.98
Dizziness	− 0.79	0.44	− 1.81	0.07
Anxiety	0.14	0.39	0.35	0.73
prEDSS	0.07	0.51	0.15	0.88
Neuro-QoL Fatigue	*− 0.27*	*0.09*	*− 2.96*	***3.49e − 3***
Beck’s Depression inventory	*− 0.23*	*0.07*	*− 3.37*	***9.43e − 4***

Significant values are in bold.

Significant values are in italics.

A stepwise backward regression approach was then applied, which involves iteratively removing the least significant variable based on the Akaike Information Criterion (AIC). The final model (Table 4) included pain, dizziness, Neuro-QoL Fatigue and BDI. The model was significant (F-statistic: 53.09, *p* < 0.001) and explained 53.0% of the variance in the Neuro-QoL Cognitive sub-score (adjusted R^2^ = 0.53).

**Table 4:** Results of the final multiple regression model after a stepwise backward regression approach with subjective performance as dependent variable.

Predictor	Estimate	Std. Error	t-value	*p*-value
Intercept	*70.75*	*3.48*	*20.33*	***< 0.001***
Pain	*− 1.52*	*0.37*	*− 4.11*	***< 0.001***
Dizziness	*−* * 0.97*	*0.38*	*−* *2.55*	***0.01***
Neuro-QoL Fatigue	*− 0.27*	*0.08*	*− 3.26*	***0.001***
Beck’s Depression inventory	*−* * 0.22*	*0.06*	*−* *3.74*	***< 0.001***

Significant values are in bold.

Significant values are in italics.

#### Objective cognitive performance

We used a multiple linear regression model to investigate the relationship between the dependent variable objective cognitive performance (Symbol test score), and independent variables related to MS symptoms (SymptoMScreen: walking, hand function, spasticity, pain, sensory, bladder, vision, dizziness, anxiety), fatigue (Neuro-QoL Fatigue), disability (patient-reported EDSS), and depression (Beck’s depression inventory; BDI). A check for multicollinearity was performed using the variance inflation factor (VIF). The VIF values for the predictor variables were all within acceptable limits, indicating no severe multicollinearity issues.

The initial model (see Table 5) included all the predictor variables, and showed that only patient-reported walking had a statistically significant negative association with the Symbol test score (β = -1.66, *p* = 0.008).

**Table 5:** Results of multiple regression model with objective cognitive performance as dependent variable.

Predictor	Estimate	Std. Error	t-value	*p*-value
Intercept	32.03	3.92	8.18	< ***0.001***
Walking	*− 1.66*	*0.62*	*− 2.67*	***0.008***
Hand function	0.70	0.56	1.25	0.21
Spasticity	0.18	0.52	0.36	0.72
Pain	− 0.40	0.49	− 0.81	0.42
Sensory	0.54	0.45	1.22	0.23
Bladder	− 0.14	0.40	− 0.36	0.72
Vision	− 0.30	0.39	-0.77	0.44
Dizziness	0.04	0.46	0.08	0.93
Anxiety	− 0.45	0.41	− 1.09	0.28
prEDSS	− 0.12	0.54	− 0.24	0.81
Neuro-QoL Fatigue	− 0.10	0.10	− 1.06	0.29
Beck’s Depression inventory	0.10	0.07	1.33	0.18

Significant values are in bold.

Significant values are in italics.

The stepwise backward regression approach resulted in a final model (see Table 6) that included walking, hand function, and vision, with walking being a significant negative predictor (β = -1.89, *p* < 0.001). This model was significant in predicting objective cognitive performance (F-statistic = 9.23, *p* < 0.001), and explained 11.8% of the variance in the dependent variable (adjusted R^2^ = 0.118).

**Table 6:** Results of the final multiple regression model after a stepwise backward regression approach with objective cognitive performance as dependent variable.

Predictor	Estimate	Std. Error	t-value	*p*-value
Intercept	*27.96*	*1.11*	*25.09*	***< 0.001***
Walking	*− 1.89*	*0.46*	*− 4.09*	***< 0.001***
Hand function	0.84	0.50	1.71	0.09
Vision	− 0.49	0.32	− 1.51	0.12

Significant values are in bold.

Significant values are in italics.

### Brain MRI volumes

A total of 78 MRI scans were uploaded to the web portal. Of these scans, 72 were brain MRI scans, and 52 were from patients who had completed app data. All scans were manually checked for adequate scan quality.

#### Subjective cognitive performance

The Neuro-QoL subjective cognition did not correlate significantly with any of the volumetric brain MRI parameters. Besides a negative non-significant correlation with CSF volume (rho = -0.36, *p* = 0.33), all other calculated correlations resulted in a rho < 0.25 and *p* > 0.90.

#### Objective cognitive performance

**Fig. 2:**
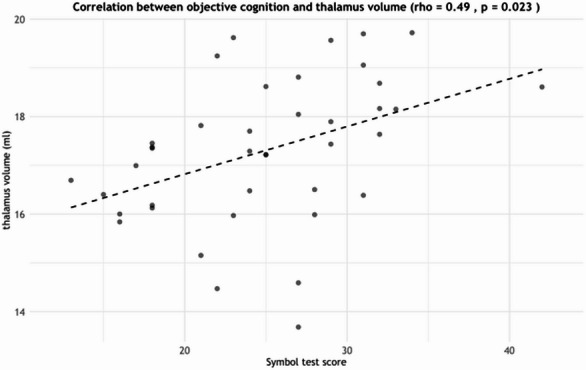
Correlation plot illustrating the relationship between thalamic MRI volume, calculated using icobrain, and objective cognitive performance as assessed by the Symbol test.

**Fig. 3:**
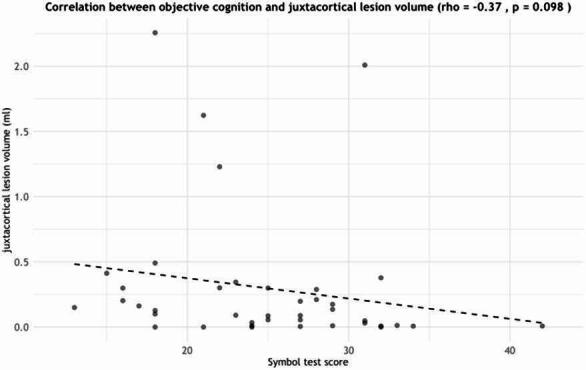
Correlation plot illustrating the relationship between juxtracortical lesion MRI volume, calculated using icobrain, and objective cognitive performance as assessed by the Symbol test.

**Fig. 4:**
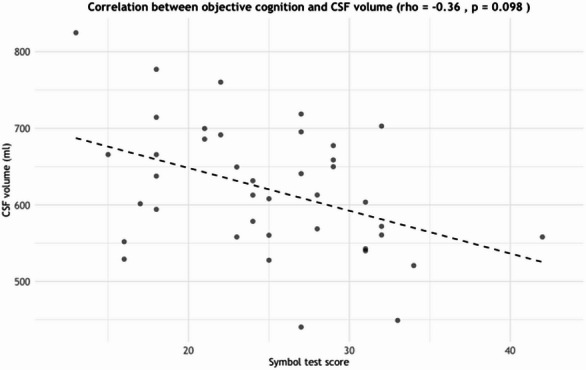
Correlation plot illustrating the relationship between cerebrospinal fluid (CSF) MRI volume, calculated using icobrain, and objective cognitive performance as assessed by the Symbol test.

**Fig. 5:**
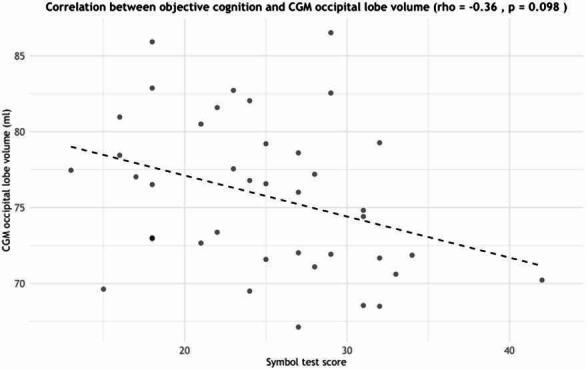
Correlation plot illustrating the relationship between occipital lobe cortical grey matter (CGM) MRI volume, calculated using icobrain, and objective cognitive performance as assessed by the Symbol test.

**Fig. 6:**
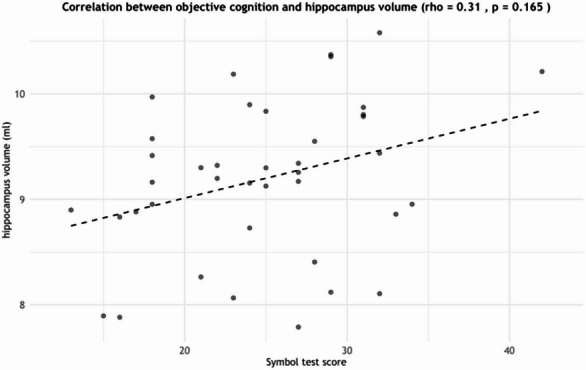
Correlation plot illustrating the relationship between hippocampal MRI volume, calculated using icobrain, and objective cognitive performance as assessed by the Symbol test.

The Symbol test correlated significantly with thalamic volume (rho = 0.49, *p* = 0.023). Juxtacortical lesions (rho = -0.37, *p* = 0.098), CSF (rho = -0.36, *p* = 0.098), occipital lobe cortical grey matter (rho = -0.36, *p* = 0.098), hippocampus (rho = 0.31, *p* = 0.165) volume and other volumetric parameters (rho > 0.25, *p* > 0.300) did not correlate significantly with objective cognitive performance (Figs. 2, 3, 4, 5 and 6).

## Discussion

The goal of this study was to identify the difference in MS symptoms and brain MRI volumes related to subjective and objective cognitive performance. We found a weak positive correlation between subjective and objective performance. Furthermore, pain, dizziness, fatigue and depression significantly and negatively predicted subjective cognitive performance, while objective scores were only negatively predicted by patient-reported walking impairment. Consistent with previous research, objective performance, as measured by a processing speed task, was significantly and positively correlated with thalamic volume in our sample. Additionally, objective performance exhibited weak non-significant relationships with hippocampal volume, juxtacortical lesions, cerebrospinal fluid (CSF) volume, and occipital lobe cortical grey matter. Notably, subjective performance did not correlate with any imaging volumetric measures.

The weak association between subjective and objective cognitive performance is in line with previous findings^8,13,14,16–23^, and several studies have shown that patients with MS often struggle to accurately assess their own cognitive status^14,16–18,20,22,23^. However, our confusion matrix shows that relying solely on subjective reporting of cognitive performance leads to 84.5% of all participants being correctly identified as cognitively impaired or non-impaired (as defined by the objective test). 8% of subjects would not be identified as cognitively impaired despite being objectively impaired according to the SDMT, and 7.5% of the sample demonstrated impaired subjective cognitive performance without any observable impairment in objective cognitive performance. This corresponds to a sensitivity of 20% and a specificity of 92%, which is in contrast with the relatively high sensitivity and low sensitivity described in previous studies^16,18^. A possible explanation could be that we only included patients with a disease duration of less than 15 years, which may suggest that patients are less likely to underestimate their cognitive performance in the earlier stages of the disease. Other studies have found that underestimation is related to depression^14,23^ and fatigue^23^, which become more prevalent with increasing disease duration^41,42^. Conversely, sensitivity was very poor, suggesting that many patients are not reporting cognitive deficits early in the disease. It is possible that the questions included in the Neuro-QoL cognitive subscore are not representative of the cognitive symptoms that patients experience in the early disease stages. In general, our findings suggest that a negative subjective cognitive screen in an early MS patient is very likely related to an absence of impairment on an objective cognitive screen, although this needs to be confirmed in future studies.

When examining the relationships between subjective and objective cognitive performance and other MS-related symptoms, the difference between the two becomes clear. In our sample, only patient-reported walking impairment predicted objective cognitive performance. This is in line with previous studies in MS showing the link between processing speed and both walking speed^43–45^ and patient-reported walking impairment^45^. In contrast, subjective cognitive performance was more broadly associated with ‘invisible’ symptoms such as pain, dizziness, fatigue and depression. This is in line with the described association of subjective performance with depression^8,13,14,16–23^ and fatigue^16,23,24^. These associations may be explained by the items questioned in the cognitive questionnaire, which may also be caused by fatigue, pain or depression and their treatments (for example slowed thinking, trouble concentrating). The link with dizziness is less straightforward, but may be related to a general poor perception of quality of life or self-worth. Indeed, all included MS symptoms only explained 11.8% of the variance in our model for objective cognitive performance, compared to 53% in the model to predict subjective performance. This suggests that the patient-reported measures are much more strongly linked to each other, and may significantly overlap with each other.

Examining the relationships between regional brain volumes calculated using icobrain and cognitive performance, we did not find significant correlations between subjective cognitive performance and brain volumes. This contrasts the previously described associations of subjective impairment and reduced thalamic and cortical grey matter volumes^11^ and reduced lower total hippocampal and CA1 volumes^26^. On the other hand, objective performance correlated significantly with thalamic volume. Additionally, we observed weak non-significant correlations between objective performance and hippocampal volume, juxtacortical lesions, cerebrospinal fluid (CSF), and occipital lobe cortical grey matter. The association between objective cognitive impairment and measures of brain atrophy is well-established^46–50^, in particular for information processing speed and thalamus volumes in patients with MS^51,52^ as well as healthy subjects^53^. The disparity of our findings in subjective and objective performance confirms that these tests seem to capture different aspects of the disease.

While objective cognitive performance is related to factors that are visible and measurable (walking, thalamic volume), subjective performance is also related to less tangible symptoms that are difficult to quantify. There is a growing interest in the impact of these invisible symptoms, which can include fatigue, depression, anxiety, cognitive impairment, pain, spasticity, sensory impairment, dizziness, bowel or bladder dysfunction, sexual dysfunction, and visual impairment. These symptoms are very common but often un(der)acknowledged by the patient’s family, friends, co-workers and health care providers^54,55^. Patients describe their symptoms being invisible or undetectable as one of the most distressing parts of having MS^55^. Moreover, these symptoms have been found to cause more health distress than visible symptoms^56^ and negatively impact patients’ social life, close relationships, leisure activities^57^, health perceptions^58,59^, health distress^59^ and quality of life^59,60^. Although patient-reported cognitive measures remain a poor substitute for an objective cognitive screening, we argue that the presence of cognitive symptoms (directly reported by the patient or measured using a subjective questionnaire) should always be taken seriously, since this is often related to other easily missed, invisible MS symptoms. The overlap between different patient-reported outcome measures may indeed be a strength, allowing clinicians to flag a broader issue, such as a poor perceived quality of life.

To the best of our knowledge, we are the first study to compare the different influencing factors of subjective and objective cognitive performance. We included a large multicentric and international sample of 205 participants, resulting in a diverse study population and increased generalizability. Furthermore, the remote data collection allowed for a ‘real-world’ assessment outside of the hospital setting. However, while 300 participants signed the informed consent form, only 205 completed the planned assessments, due to the inherent lack of oversight in remote evaluations. Another limitation of this study is that only a quarter of the included participants had an available MRI measurement. Moreover, the level of disability was low, probably in part due to the exclusion of patients with a disease duration of longer than 15 years, which limits our conclusions to patients with early MS. Furthermore, as is inherent to remote studies using digital applications, patients with severe dexterity or cognitive limitations are automatically excluded from participation. Our objective cognitive evaluation was limited to only one test, which was designed to assess only one cognitive domain. This choice was made in analogy with the recommendations for cognitive screening in MS patients (SDMT^5^), but it limits the generalisability of our findings to other cognitive domains. Additionally, only 10% of our subjects was impaired based on this test, which is significantly lower than the described population prevalence^2^. A limitation of the subjective cognitive evaluation and the other symptom questionnaires is that patient-reported questionnaires often ask about changes compared to baseline, which is not known in this sample and thus does not allow us to take the premorbid status into account. Lastly, we acknowledge the ongoing debate on dichotomising scores in multiple sclerosis, which might reduce statistical power^61,62^. We nonetheless deemed it justified as an additional way to analyse our data, clarifying the overlap between subjective and objective cognitive performance.

Future studies should include patients with a more widespread distribution of disease durations, to allow for assessment of the role of subjective cognitive measures as cognitive screening tools in early versus later stages of the disease. Other studies could also look at the relationships of objective and subjective cognitive performance with other more ‘measurable’ clinical markers such as walking speed and balance or more advanced imaging biomarkers such as diffusion-weighted imaging or functional MRI. Additionally, exploring psychological variables such as perceived quality of life or self-worth could provide insights into the subjective cognitive component, particularly in the absence of objective impairment.

This study provides new insights into the distinct yet complementary roles of subjective and objective cognitive performance in individuals with MS. Subjective cognitive performance is closely linked to ‘invisible’ symptoms such as pain, fatigue, dizziness, and depression, while objective cognitive performance correlates more strongly with measurable clinical markers, such as thalamic volume and walking impairment. These findings highlight the need to consider both subjective and objective cognitive performance when treating an MS patient, as these are related to a different set of clinical markers. A more nuanced approach that integrates both perspectives could improve clinical assessments and enable more tailored therapeutic interventions, ultimately contributing to holistic and patient-centred care.

## Data Availability

The datasets used and/or analysed during the current study are available from the corresponding author upon reasonable request.
